# Is a history of cesarean section a predictive factor for the failure of medical management in spontaneous abortion?

**DOI:** 10.1007/s00404-026-08504-1

**Published:** 2026-06-27

**Authors:** Ravit Pertez-Machluf, Maya Youran Kimhi, Rotem Yedidia Moser, Nissim Arbib, Yair Daykan, Omer Weitzner, Ron Schonman, Zvi Klein, Yael Yagur

**Affiliations:** 1 Department of Obstetrics and Gynecology, Sheba Medical Center, Tel Hashomer, Ramat Gan, Israel; 2 Department of Obstetrics and Gynecology, Meir Medical Center, Kfar Saba, Israel; 3 School of Medicine, Gray Faculty of Medical and Health Sciences, Tel Aviv University, Tel Aviv, Israel; 4 Rappaport Faculty of Medicine, Technion-Israel Institute of Technology, Haifa, Israel

**Keywords:** Cesarean delivery (CD), Medical abortion, Retained products of conception (RPOC), Spontaneous abortion

## Abstract

**Objectives:**

This study aims to evaluate the risk factors for failure of medical treatment for first-trimester spontaneous abortion and the specific role of prior cesarean delivery (CD).

**Methods:**

This retrospective cohort study analyzed data from patients who underwent medical management of first-trimester spontaneous abortion using mifepristone and misoprostol at a tertiary medical center between 2019 and 2022. Data collected included demographic, clinical, and sonographic parameters, with comparisons made between patients with successful and unsuccessful treatment outcomes. Univariable and multivariable logistic regression analyses were conducted.

**Results:**

Among the 716 patients included in the study, treatment failure was observed in 29.5%. In multivariable analysis, prior CD was independently associated with an increased risk of treatment failure (aOR 1.97; 95%CI 1.25–3.12; *p* = 0.004). Additionally, advanced gestational age was also associated with an increased likelihood of failure (aOR 1.02 per day; 95%CI 1.00–1.03; *p *= 0.009). In subgroup analyses, patients with prior CD had higher failure rates compared with those without prior CD (43.2% vs 27.4%, *p* = 0.002). Among patients without prior CD, gestational age > 70 days was associated with higher failure rates compared with ≤ 70 days (35.2% vs 24.3%, *p *= 0.006). In this subgroup (no prior CD and gestational age ≤ 70 days), the presence of bleeding during treatment was associated with lower failure rates (12.5% vs 25.6%, *p* = 0.001).

**Conclusion:**

Prior cesarean delivery was independently associated with an increased risk of treatment failure following medical management of first-trimester spontaneous abortion. Advanced gestational age was also associated with increased failure rates. Further studies are needed to refine treatment protocols and explore approaches for patients with prior CD.

## What does this study add to the clinical work?

**Table:** 

Women with a history of caesarean delivery experienced higher rates of treatment failure following medical management of first-trimester spontaneous abortion. Prior uterine scarring should be considered when evaluating factors associated with treatment outcomes.	

## Introduction

Spontaneous abortion in the first trimester affects approximately 10% of confirmed pregnancies, a frequent concern for both patients and healthcare providers [1]. Management options include expectant, surgical, and pharmacological interventions. The pharmacological treatment includes misoprostol, often in combination with mifepristone, with reported success rate, between 80 and 90% depending upon the definition used [2–4]. Misoprostol can be administered via oral, vaginal, sublingual, or buccal routes. The addition of mifepristone, an anti-progesterone agent, enhances uterine receptivity to misoprostol [5]. This combination has been shown to improve overall success rates of the treatment in multiple studies [2, 6–8]. The recommended choice of the administration route depends on clinical protocols and patient preferences [6, 9–12].

Predictors of success have been extensively explored [13–15], yet data are limited regarding the influence of a history of cesarean section or other uterine surgery on the effectiveness of pharmacological interventions [16–18].

Cesarean delivery (CD) is one of the most commonly performed surgical procedures worldwide, with rates ranging from 20 to 50% depending upon the population [19]. CD is associated with long-term uterine scarring. Uterine scar tissue, by altering endometrial receptivity and myometrial contractility [20], can thereby diminish the efficacy of medications and scarring may create anatomical barriers that hinder proper expulsion of gestational tissue [16].

Data on the specific relationship between prior CD and the success rates of medical management for first-trimester missed abortion remain limited and inconsistent [16–18]. Understanding this relationship is critical to optimizing treatment protocols for women with this surgical history.

This study seeks to bridge the knowledge gap by identifying risk factors associated with treatment failure in first-trimester miscarriages, with a particular focus on how a history of CD influences the success rates of mifepristone misoprostol therapy.

## Methods

### Study population

This retrospective cohort study included patients treated for spontaneous abortion at a tertiary medical center between January 2019 and February 2022. Patients diagnosed with first trimester (≤ 12 week and 6 day gestational age) with spontaneous abortion who received pharmacological management with mifepristone and misoprostol were included. Exclusion criteria included multiple pregnancies, the presence of intrauterine devices, or deviations from the standard protocol.

### Outcomes

The primary outcome was to identify risk factors associated with treatment failure of mifepristone/misoprostol treatment in first-trimester spontaneous abortions including the role of prior cesarean deliveries and how they influence treatment outcomes.

### Treatment protocol

Patients diagnosed with spontaneous abortion were provided with options for either medical or surgical management in accordance with clinical guidelines and their preferences. Patients opting for medical treatment first received 200 mg of mifepristone administered orally under medical supervision. Following this, they were discharged with instructions to return 24 h later for the administration of 800 µg of buccal misoprostol.

One week after misoprostol administration, a clinical and sonographic evaluation was conducted in the clinic. If residual tissue with Doppler flow or an endometrial thickness exceeding 15 mm was detected, a second dose of 800 µg of misoprostol was administered. Patients were re-evaluated one week later, and those with persistent retained products of conception (RPOC) were referred for hysteroscopy and removal of RPOC. This procedure was typically scheduled 3–8 weeks after the final misoprostol dose. Treatment failure was defined as persistent sonographic evidence of RPOC at follow-up transvaginal ultrasound after completion of the medical treatment protocol. Specifically, failure was defined as an endometrial thickness > 15 mm or the presence of Doppler flow suggestive of RPOC. According to our institutional protocol, these findings prompted referral for hysteroscopic removal. This threshold was selected based on evidence supporting the clinical relevance of a 15 mm cutoff for identifying clinically significant RPOC [21, 22].

### Data collection

Data extracted from electronic medical records included demographic, obstetric, and clinical variables. Demographic information consisted of age, BMI, marital status, and smoking habits. Obstetric history encompassed gravidity, parity, prior abortions, and details of prior uterine or cervical surgical interventions, including cesarean sections. Clinical variables included the mode of conception (spontaneous or artificial), the presence of bleeding, gestational age, sonographic gestational age, delta between gestational and sonographic age, the presence of an embryonic pole, post-treatment endometrial thickness measurements, and Doppler flow.

Cases were analyzed and compared based on treatment outcomes, and categorized into success and failure groups.

### Ethics

The study was approved by the institutional review board of the tertiary medical center (MMC-#0087–16). Due to the retrospective nature of the study, informed consent was waived. Data confidentiality and anonymity were maintained throughout the research process.

### Statistical analysis

Categorical variables were summarized as frequencies and percentages, while continuous variables were described using means and standard deviations or medians and interquartile ranges. Differences between groups were analyzed using Chi-square tests or Fisher’s exact tests for categorical variables and independent *t* tests or Mann–Whitney *U* tests for continuous variables. Logistic regression analysis was performed to identify predictors of treatment failure, with adjustments for confounders, such as gestational age, parity, and mode of conception. Variables included in the model were selected based on clinical relevance and their association with the outcome in univariable analysis.

All statistical analyses were conducted using SPSS.

All statistical tests were two sided and *p* < 0.05 was considered statistically significant.

## Results

Throughout the study period, 817 patients with a diagnosis of first-trimester spontaneous abortion were directed to undergo pharmacological treatment. Among them, 20 had multiple pregnancies, and 1 was found to have an intrauterine device in place. Furthermore, 56 patients received treatment that deviated from the established protocol, and 25 were unavailable for follow-up.

Of the 716 patients included in the study, 95 (13.3%) had a history of CD, while the other 621 (86.7%) had no record of prior uterine surgeries. Treatment success was achieved in 506 patients (70.5%), whereas 211 patients (29.5%) experienced treatment failure.

Table 1 presents the baseline characteristics of the study groups. No significant differences were observed between the success and failure groups in terms of demographic parameters, including age, BMI, marital status, smoking status, gravidity, parity, history of abortion, maternal morbidities (such as autoimmune conditions, diabetes, and cardiovascular conditions), uterine or cervical manipulation, mode of conception, and bleeding.

**Table 1:** Baseline characteristics of the study groups

Characteristics	Success *N* = 505	Failure *N* = 211	*p* value
Age (years)	33.65 ± 5.73	33.63 ± 5.63	0.95
BMI (kg/m^2^)	24.85 ± 4.90	24.39 ± 5.02	0.15
Marital status			> 0.99
Single	59 (11.7%)	23 (10.9%)	
Married	436 (86.3%)	184 (87.2%)	
Divorced	10 (2.0%)	4 (1.9%)	
Smoking	28 (6.2%)	16 (8.2%)	0.34
Background conditions			
Autoimmune condition	62 (12.3%)	19 (9.0%)	0.21
Diabetes	6 (1.2%)	3 (1.4%)	0.73
Cardiovascular conditions	7 (1.4%)	2 (0.9%)	> 0.99
Coagulopathy	3 (0.6%)	4 (1.9%)	0.2
Parity	1.24 ± 1.25	1.4 ± 1.25	0.06
Prior abortion	65 (30.8%)	171 (33.9%)	0.65
Prior cesarean section	54 (10.7%)	41 (19.4%)	0.43
Prior cervix manipulation	55 (10.9%)	22 (10.4%)	0.86
Artificial mode of conception	88 (17.4%)	25 (11.8%)	0.06

Data are *n* (%) or mean ± standard deviation

*BMI* body mass index

Prior CD was more frequent in the treatment failure group compared to the success group (19.4% vs. 10.7%; respectively, *p* = 0.43).

Table 2 presents the clinical characteristics of the study groups, focusing on gestational parameters and sonographic findings. Patients in the failure group had a slightly higher mean gestational age at diagnosis compared to those in the success group (66.99 ± 9.73 days vs. 64.65 ± 10.14 days; respectively, *p* = 0.005), although no significant differences were observed in sonographic gestational age. The presence of an embryonic pole was comparable between the groups. Post-treatment sonographic findings revealed significantly higher endometrial thickness in the failure group (14.82 ± 7.5 mm vs. 10.02 ± 4.72 mm; *p* < 0.001). Additionally, the first maximal residual tissue size was significantly larger in the failure group compared to the success group (18.5 ± 10.6 mm vs. 13.4 ± 5.7 mm; *p* < 0.001).

**Table 2:** Clinical characteristics of the cohort

Characteristics	Success *N* = 505	Failure *N* = 211	*p* value
Gestational age (days)	64.65 ± 10.14	66.99 ± 9.73	0.005
Sonographic gestational age (days)	45.98 ± 8.47	47.1 ± 9.15	0.22
Delta between gestational age and ultrasound age (days)	18.67 ± 9.42	19.89 ± 10.25	0.12
Bleeding	113 (23.6%)	37 (19.3%)	0.23
Embryonic pole			
0	97 (21.1%)	38 (18.8%)	0.5
1	363 (78.9%)	164 (81.2%)	
First max endometrium (mm)	14.82 ± 7.5	10.02 ± 4.72	< 0.001
First max residual size (mm)	13.4 ± 5.7	18.5 ± 10.6	< 0.001

Data are *n* (%) or mean ± Standard deviation

Multivariable regression analysis identified prior CD as an independent predictor of treatment failure (adjusted odds ratio [aOR] 1.97; 95%CI 1.25–3.12; *p* = 0.004), corresponding to nearly double the odds of failure. Higher gestational age was also associated with an increased risk of failure (aOR 1.02; 95%CI 1.00–1.03; *p* = 0.009). No significant associations were found for mode of conception and parity (Table 3).

**Table 3:** Multivariable analysis

Characteristics	aOR	*p* value
Cesarean section	1.97 (CI 1.25–3.12)	0.004
Mode of conception	0.78 (CI 0.47–1.27)	0.31
Gestational age (days)	1.02 (CI 1.00–1.03)	0.009
Parity	1.04 (CI 0.91–1.19)	0.58

Exploratory subgroup analyses were performed to further characterize treatment failure rates across clinically relevant groups. Subgroup analysis revealed that patients with prior CD had a significantly higher failure rate (43.2%) compared to those without (27.4%), *p* = 0.002, corresponding to an absolute risk difference (ARD) = 15.8%. Additionally, among patients with no prior CD, those with a gestational age > 70 days experienced higher failure rates (35.2%) compared to those with gestational age ≤ 70 days (24.3%), *p* = 0.006, ARD = 10.9%.

**Fig. 1:**
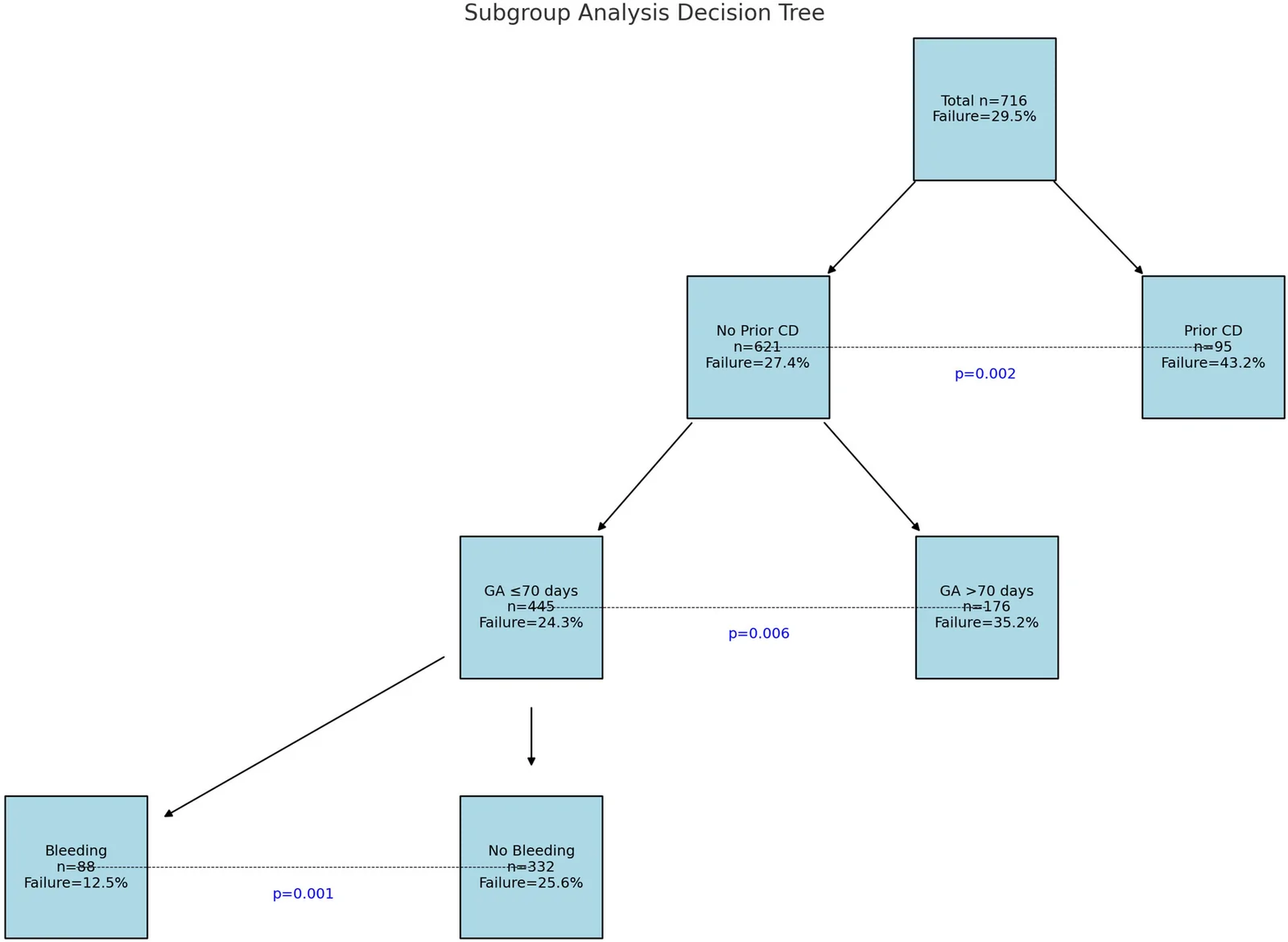
This decision tree illustrates treatment failure rates across different clinical subgroups. The initial stratification is based on the presence or absence of prior CD. Patients with a prior CD had a significantly higher failure rate (43.2%) compared to those without (27.4%, *p* = 0.002). Among patients with no prior CD, those with a gestational age (GA) > 70 days had higher failure rates (35.2%) than those with GA ≤ 70 days (24.3%), (*p* = 0.006). Within the GA ≤ 70 days group, the presence of bleeding during treatment was associated with a lower failure rate (12.5%) compared to no bleeding (25.6%), (*p* = 0.001). Dashed lines between boxes indicate statistically significant comparisons between paired groups

Among patients with no prior CD and gestational age ≤ 70 days, the presence of bleeding during treatment was associated with lower failure rates (12.5% vs. 25.6%, ARD = 13.1%, *p* = 0.001). (Fig. 1).

## Discussion

This study focused on risk factors contributing to the failure of medical management for spontaneous abortion up to 12 + 6 weeks of gestation, with specific exploration of whether a history of CD serves as a predictive factor for treatment outcomes. Our findings indicate that prior CD was independently associated with a higher likelihood of treatment failure.

The history of CD as a significant independent predictor of treatment failure aligns with prior research. One study examining artificial medical abortions included 879 patients up to 56 days of gestation. The findings revealed significantly higher odds of failure among patients with prior cesarean deliveries compared to those with no or fewer cesareans [18]. Another study supporting this finding explored the effects of cesarean scars by examining 183 patients undergoing artificial medical abortion up to 56 days of gestation. It revealed that patients with sonographic evidence of a cesarean scar defect were more likely to experience failure compared to those without such defects [23]. Those studies, however, were limited to gestational ages up to 56 days in the context of artificial abortion. Additional data indicated that patients undergoing artificial medical abortion up to and including 12 + 6 weeks of gestation with a history of prior CD were found to have an independent risk factor for failed medical abortion [16]. However, conflicting evidence exists, highlighting the need for further exploration. Dehlendorf et al. included 1690 patients undergoing medical management for spontaneous abortion up to 63 gestational days. Among these, 200 patients with a history of CD had an odds ratio of 1.8 for treatment failure, but the result was not statistically significant [17].

One reason for this finding may be related to anatomical changes. Uterine scarring from the previous surgeries may be associated with uterine contractility which could potentially influence the effectiveness of misoprostol. Moreover, such scarring may disrupt normal endometrial receptivity, leading to altered implantation patterns in subsequent pregnancies and contributing to reduced expulsion efficiency during medical abortion [24, 25].

Another important finding was that increased gestational age significantly correlates with higher rates of treatment failure. Specifically, a subanalysis focused on patients without prior CD, which demonstrated that those with a gestational age greater than 70 days had failure rates of 35.2%, compared to 24.3% for those with a gestational age of ≤ 70 days. Furthermore, in those patients with gestational age ≤ 70 days, the presence of bleeding during treatment was associated with lower failure rates. Support for this contention exists in the medical literature. Medication abortion failure increases with advancing gestational age through 70 days of gestation [5, 26]. Furthermore, studies focused on a later gestational age demonstrated that the success rate at 71–77 days was 86.7%, significantly lower than the rate at 64–70 days, which was 92.3% [27]. Meaidi et al. reported that the proportion of surgical interventions increased from 3.5% in the 5th-to-6th gestational week to 10.3% in the 9th week [28]. Similarly, Chien et al. found that gestational ages greater than 42 days were associated with higher odds of failure [18]. This suggests that as gestational age advances, structural and physiological changes, such as greater trophoblastic invasion and enhanced placental attachment, may contribute to reduced efficacy of medical abortion protocols as well as altered hormonal levels, thus reducing the responsiveness to pharmacological agents. Further evidence supports bleeding as a contributing factor in the reduction of failed medical management. Studies show that early onset and resolution of bleeding are associated with successful expulsion without surgical intervention [29, 30].

Sonographic findings are integral to predicting treatment failure. In our study, increased endometrial thickness one week after administering a single dose of misoprostol, following mifepristone, was significantly associated with treatment failure. According to our protocol, patients with an endometrial thickness greater than 15 mm or suspected RPOC with Doppler flow detected on follow-up ultrasound were administered a second dose of 800 µg of misoprostol. These sonographic parameters provide a basis for identifying individuals at higher risk of RPOC and guiding subsequent clinical interventions. The American College of Obstetricians and Gynecologists (ACOG) recommends the option of a single repeat dose in cases of incomplete uterine evacuation [1, 5]. However, there is no agreement across studies regarding the criteria for timing of second dose and determining complete evacuation of pregnancy tissue when a gestational sac is absent, as varying thresholds of endometrial thickness have been reported. Additionally, the efficacy of administering a second dose of misoprostol in patients with a thickened endometrium remains uncertain [31, 32]. In our cohort, increased endometrial thickness after the first misoprostol dose was associated with higher rates of treatment failure. This uncertainty stems from the lack of clarity regarding the optimal timing, dosage, and effectiveness of a second misoprostol administration at different gestational ages. Consequently, when a thickened endometrium and Doppler flows suggest RPOC, the likelihood of failure rises. Moreover, our protocol is stricter than the broad definitions of success, which rely solely on the absence of a gestational sac. We require not only an endometrial thickness of less than 15 mm but also the absence of any sonographic suspicion of RPOC. Recognizing that hysteroscopy is a low-risk intervention and a crucial tool for assessing uterine integrity, especially for future fertility, we adopt a more proactive approach. When necessary, we opt for diagnostic hysteroscopy to ensure the completeness of treatment and the optimal long-term reproductive outcomes for our patients [33]. While this approach may increase the proportion of patients classified as treatment failures compared to studies using less stringent criteria, it reflects a deliberate clinical choice. By applying a stricter sonographic threshold, RPOC can be detected at an earlier stage, thereby preventing chronic complications, such as intrauterine adhesions, prolonged bleeding, or infection [34–36]. This strategy is designed to preserve uterine integrity and optimize future fertility, especially in women with prior uterine surgery who may be at higher risk. This proactive approach may support better long-term outcomes, even if it results in a higher short-term intervention rate.

Based on our findings and the current literature, we recommend a nuanced approach to the medical management of first-trimester miscarriage. While our protocol adopts a relatively strict sonographic threshold (> 15 mm endometrial thickness) for defining treatment failure, we recognize that immediate surgical intervention may not always be necessary, especially in asymptomatic women. In line with the recent international guidelines, we suggest that a short period of expectant management up to 30 days can be considered for patients with persistent thickened endometrium, as long as there are no signs of infection or excessive bleeding. For women with additional risk factors such as prior CD or advanced gestational age, closer follow-up and a lower threshold for intervention may be warranted. Ultimately, management decisions should, therefore, be individualized and based on clinical symptoms, patient preference, and follow-up findings. Further studies are needed to validate the optimal timing and criteria for surgical intervention in this setting and to ensure the best long-term outcomes.

This study has several strengths. First, it addresses an unresolved clinical question by focusing on the impact of prior CD on the outcomes of medical spontaneous abortion. The large sample size and inclusion of a well-defined cohort provide statistical power. Additionally, the study’s use of standardized treatment protocols and detailed sonographic follow-up assessments allows for consistent evaluation of outcomes. Another strength of our study lies in its strict definition of treatment failure, by defining failure not only as an endometrial thickness above 15 mm and including cases with Doppler-detected RPOC even with endometrial thickness below 15 mm. This approach aims to ensure that no remnants are left, as RPOC can increase the risk of infertility and negatively impact future reproductive health. Finally, the study’s findings are actionable, as they offer an opportunity to refine the existing protocols and management strategies particularly in the context of prior surgical history and gestational age.

Despite its strengths, the study is not without limitations. Its retrospective design introduces potential biases, as it relies on the existing medical records. The single-center setting and the specific follow-up protocol, which relies on ultrasound for the evaluation of success, may limit the generalizability of the findings. Furthermore, the strict and conservative definition of treatment failure used in this study, which includes cases with Doppler-detected RPOC even when the endometrial thickness is below 15 mm, may lead to a higher reported failure rate compared to studies using more lenient criteria. Our institutional protocol prioritizes complete evacuation to prevent long-term complications such as infertility, which drives our approach to intervene with hysteroscopy more aggressively when sonographic findings are suspicious. While this strategy ensures a high degree of uterine cavity clearance, it may overestimate failure rates when compared to protocols that define success simply as the absence of a gestational sac.

## Conclusions

This study found that prior CD was independently associated with a higher risk of failure in the medical management of first-trimester spontaneous abortion, with failure rates also increasing with advancing gestational age. These findings underscore the need to further investigate individualized treatment thresholds.

## Data Availability

Data can be available upon reasonable request from the corresponding author.
